# A biopsychosocial-cultural model for understanding oral-health-related quality of life among adolescent orthodontic patients

**DOI:** 10.1186/s12955-020-01334-y

**Published:** 2020-03-30

**Authors:** Hua Ao, Xiao Deng, Ying She, Xin Wen, Qingrong Wu, Fuguo Chen, Xiao Gao

**Affiliations:** 1 Faculty of Psychology, Southwest University, #2 Tiansheng Road, Beibei District, Chongqing, China; 2 Key Laboratory of Cognition and Personality, Southwest University, Chongqing, China; 3 Department of Orthodontics, Stomatological Hospital of Chongqing Medical University, Chongqing, China; 4 Chongqing Municipal Key Laboratory of Oral Biomedical Engineering of Higher Education, Chongqing, China

**Keywords:** Oral-health-related quality of life, Orthodontics, Biopsychosocial-cultural model, Adolescents, Social reinforcement, Dental aesthetics

## Abstract

**Background:**

Based on previous theoretical oral-health-related quality of life (OHRQoL) models and most recently framework, as well as sociocultural model of body image dissatisfaction, the current study aimed to investigate the effect of individual (dental aesthetics and dental appearance social comparison) and sociocultural factors (social reinforcement from parents, peers and mass media on dental aesthetics) as well as their interaction on psychosocial dimension of OHRQoL among adolescent orthodontic patients.

**Methods:**

In this cross-sectional study comprising 427 adolescent orthodontic patients (151 boys and 276 girls) aged between 11 and 16 years old, the psychosocial dimension of OHRQoL was measured by Psychosocial Impact of Dental Aesthetics Questionnaire. Individual predictor of dental aesthetics was defined by the Aesthetic Component of the Index of Orthodontic Treatment Need, and dental appearance social comparison was assessed by four items adapted from Physical Appearance Comparison Scale. Sociocultural predictor of social reinforcement was measured by six items adapted from Perceived Sociocultural Pressure Scale. Spearman correlations, path analyses, and structural equation modeling were used to build up several predictive models.

**Results:**

As hypothesized, two direct pathways were observed that patients’ dental aesthetics and all three sources of social reinforcement directly predicted the psychosocial dimension of OHRQoL. Meanwhile, we observed one indirect pathway, that three sources of social reinforcement predicted the psychosocial dimension of OHRQoL, in part, through dental appearance social comparison.

**Conclusions:**

This study provides preliminary evidence indicating that dental aesthetics, social reinforcement and dental appearance comparison are reliable predictors of psychosocial dimension of OHRQoL among adolescent orthodontic patients.

## Background

Both theoretical models [1–3] and evidence-based research [4–6] characterized oral-health-related quality of life (OHRQoL) as a multidimensional construct. Sociocultural influence is an important dimension in most OHRQoL models [3, 7, 8]. Recently, Gupta et al. postulated a new detailed framework for understanding how social (social economic status and social network) and psychological factors (e.g., sense of coherence, social support and stress) interacted on OHRQoL outcomes in adult patients [9], which gave support for the psychosocial-cultural pathway being one of the key factors on OHRQoL.

Yet, to date, little is known about the effects of interactions between sociocultural and individual factors (both biological and psychological) on OHRQoL among adolescent orthodontic patients. Although previous models provided some detailed framework [7–9], orthodontic-specific OHRQoL and generalization from adults to children/adolescents needs further considerations. Growing evidence has shown that improved dental aesthetics, self-confidence and social interaction are important orthodontic treatment benefit [10–12]. In most cases, to improve dental aesthetics is one of the main reasons for seeking orthodontic treatment [13, 14]. Thus, dental aesthetics and its psychosocial impact constitute the key dimensions of orthodontic-specific OHRQoL. Further, how sociocultural factors exerting their influence on oral health outcome is sharply different between adult and adolescent populations. For example, children’s social network and their social reinforcement mainly involves their interaction with parents, peers and the mass media. Whereas, the influence of social interaction with parents become less prominent among adults.

Social reinforcement refers to the process whereby people internalize definitions and exhibit behaviors and values approved of by significant others [15]. Applied to the sociocultural dimension of orthodontic specific OHRQoL, social reinforcement would be defined as comments or actions of significant others that serve to support and perpetuate a good-looking dental appearance. For example, if an adolescent’s peer group laughs at him/her about his/her dental appearance, if he/she receives negative comments from his/her parents on dental appearance, or if he/she notices the pressure to improve his/her dental appearance from mass media, he/she may be more likely to concern with his/her dental appearance, which may in turn increase the likelihood of poor OHRQoL in the psychosocial dimension (e.g. low dental self-confident, self-handicapping behaviors in social interaction, and so on).

Indeed, social reinforcement from parents, peers and the mass media has been found to be key processes to shape up adolescents’ maladaptive attitude, emotion and behaviors in appearance and body image domain [16–19]. Appearance pressure from peers and parents (e.g., teasing, exclusion, negative comments and ignorance) resulted in weight concern among adolescents [20]. Also, perceived pressure to be thin from the mass media could predict body dissatisfaction [21]. Furthermore, social reinforcement was also found to interact with individual’s biological conditions (e.g., body mass index) and psychological characters (e.g., low self-esteem and appearance social comparison) to promote body image disturbance or eating disorders [22–27]. However, few study has investigated the effects of social reinforcement on orthodontic OHRQoL.

Accordingly, the current study aimed to investigate the effects of individual factors (biological: dental aesthetics; psychological: dental appearance social comparison) and sociocultural factors (social reinforcement from parents, peers and mass media on dental aesthetics) as well as their interaction on psychosocial dimension of OHRQoL (psychosocial impact of dental aesthetics, PIDA) among adolescent orthodontic patients. Based on previous theoretical OHRQoL models [3, 7–9], as well as sociocultural model of body image dissatisfaction [22–27], it was hypothesized to observe that:

- dental aesthetics and social reinforcement (from parents, peers and mass media) would directly predict PIDA; and
- social reinforcement would also exert its impact on PIDA through dental appearance social comparison.

## Methods

The current study was approved by the ethical review board of the Stomatological Hospital of Chongqing Medical University, China. Before each assessment, an assent form was obtained from the subject and additional signed consent form was obtained from one of the subject’s parents.

A cross-sectional design was used in the current study. All subjects were recruited from Stomatological Hospital of Chongqing Medical University in Chongqing, China. Adolescence is generally identified as beginning with the middle school years and ending at around 18 years [28]. All adolescent patients who were about to receive labial orthodontic treatment with fixed appliances were included in the current study. The inclusion criterions were as follows: currently studying in a middle school, with no history of orthodontic treatment, no past/current neurological or psychiatric illness. Thirty-eight patients refused to take part, and 20 questionnaires were invalid (30% items of one questionnaire were missed or most items were filled with the same answers). Therefore, the final sample included 427 subjects (151 boys, 276 girls). Post-hoc power analysis was conducted by Gpower version 3.1, using the lowest coefficient of determine found in this study (*R*^2^ = 0.03). It revealed that the power was 0.95, indicating the present sample size was enough.

### Measurements

#### Criteria variable: psychosocial impact of dental aesthetics

Psychosocial Impact of Dental Aesthetics Questionnaire (PIDAQ) were used to measure the criteria variable [29]. This 23-item scale assesses psychosocial impact of dental aesthetics in young adults and adolescents. It consists of four subscales representing dental self-confidence (PIDAQ-DSC), aesthetic concerns (PIDAQ-AC), psychological impact (PIDAQ-PI) and social impact (PIDAQ-SI). Each item was rated on a five-point Likert scale, ranging from 0 = *not at all* to 4 = *very strongly*. To ensure the same direction of scoring for all items of the questionnaire, PIDAQ-DSC were scored reversely to produce a consistent measure of the impacts. Items for all of the four subscales were averaged. In the current study, Cronbach’s *α* coefficients were 0.90 for PIDAQ-DSC, 0.75 for PIDAQ-AC, 0.83 for PIDAQ-PI and 0.85 for PIDAQ-SI.

#### Predictor variables

##### Dental aesthetics

Aesthetic Component of the Index of Orthodontic Treatment Need (IOTN-AC) were used to measure patients’ dental aesthetics [30]. It presents 10 black and white photographs of anterior teeth displaying varying degrees of malocclusion, arranged from number 1 = *most attractive*, to 10 = *least attractive*. Two experienced orthodontists evaluated patients’ dental aesthetics by choosing one photograph from this scale that was most similar with patient’s dental appearance, and both of whom were blind to the patients when they did the ratings. Patients’ dental appearance was presented by their photos taken at their first visit before treatment. These two experienced orthodontists received training in the West China College of Stomatology at Sichuan University. The calibration protocol was similar as the one reported previously [31]. The kappa coefficient was 0.88 between the two orthodontists.

##### Social reinforcement on dental aesthetics

Social reinforcement on dental aesthetics from parents, peers and mass media was examined with six items adapted from Perceived Sociocultural Pressure Scale (PSPS) [17]. Items have been modified slightly to reflect pressure to improve dental appearance. Two items for each source assessed perceived pressure to have good looking teeth (e.g., I’ve perceived a strong message from my parents to have good looking teeth). Subjects responded on a 5-point Likert scale ranging from *1* = *none* to *5 = a lot.* Cronbach’s *α* coefficients were 0.88 for the parents subscale, 0.84 for the peer subscale, and 0.85 for the media subscale in the current sample.

#### Mediating variables

##### Dental appearance social comparison

Four items adapted from Physical Appearance Comparison Scale (PACS) were used to measure social comparison on one’s dental appearance (e.g., “At parties and other social occasions I compare my dental appearance to that of others”) [32]. Subjects responded on a 5-point Likert scale from *1 = never* to *5 = always*. Scores were averaged to form the appearance comparison variable, with higher scores indicating higher level of social comparison on teeth. In the current sample, it has adequate internal consistency with Cronbach’s *α* = 0.80.

#### Sociodemographic information

Age, gender, ethnicity (Han vs. minorities), parental education, household income, height and weight were queried.

### Procedure

Data collection occurred at Stomatological Hospital of Chongqing Medical University in China during June 2013 to December 2015. Once a patient started orthodontic treatment, a trained orthodontist explained the aim of this study to him/her. After the consent form were obtained, data were collected through face-to-face interviews at the same hospital by one of two trained graduate students. During the interview, the graduate student firstly introduced and explained the questionnaires to the patients, and then patients filled in all of the self-completed questionnaires which took about 40 min. PIDAQ, PSPS and PASC have been translated previously into Chinese and back-translated into English by two Ph.D. candidates in English at Southwest University in Chongqing to ensure item meanings were as originally intended. IOTN-AC was filled by two experienced orthodontists for patients.

### Data analysis

Data analysis was performed using the Statistical Package for the Social Sciences (SPSS, version 16.0, Chicago) and AMOS 24. Mean imputation was used to deal with missing data. Preliminary analyses included the evaluation of missing data, comparisons of results based on casewise deletion versus missing-values imputation, and evaluations of multi collinearity. Next, gender and household income differences in PIDAQ, IOTN-AC, PSPS and PACS were assessed using one-way ANOVA, followed by Spearman correlation analyses between age and research measures. Specifically, gender differences were conducted by comparing these research measures between girls and boys. Then, patients were divided into four groups based on their monthly household income, namely Less than 3000 RMB, 3000–5000 RMB, 5000–10,000 RMB and More than 10,000 RMB, and household income differences were compared among these groups.

Main analyses included three steps. First, Spearman correlation analyses were carried out between all the criteria, mediating and predicting variables. Second, based on the results of the first step, path analyses outlined by Baron and Kenny was used to test the mediating pathway [33]. The potential pathways included (1) parents social reinforcement-dental appearance social comparison-PIDA; (2) peers social reinforcement-dental appearance social comparison-PIDA; and (3) media social reinforcement-dental appearance social comparison-PIDA. Linear regressions were to assess whether all conditions have been met for a variable to be a mediator. Sobel tests were conducted to examine the significance of the indirect effects of the mediators [34]. In the last step of main analyses, structural equation modeling (SEM) through the method of maximum likelihood were conducted to build up a model for understanding how those biological and sociocultural predictors interacted with psychological factors on PIDA. The indices used to assess the goodness of fit of the model included the ratio of chi-square by degrees of freedom (*χ*^2^/*df*), Akaike information criterion (AIC), comparative fit index (CFI), and root mean square error of approximation (RMSEA) [35]. The fit of the models was considered adequate when *χ*^2^/*df* ≤ 2.0, CFI ≥ 0.90, and RMSEA ≤0.05 [27].

## Results

### Preliminary analysis

The entire sample (*N* = 427) was comprised of 151 boys and 276 girls between 11 and 16 years old (M = 13.96 years, SD =1.88 years). Descriptive statistics for the variables of interest are shown in Table 1. Preliminary analyses showed that girls reported significant higher scores than boys on PIDAQ, social reinforcement from parents and peers, and dental appearance social comparison. More household income was associated with higher scores on PIDAQ and all sources of social reinforcement. Age significantly correlated with PIDAQ scores (*r* = 0.12, *p* = 0.018). We did not observe any demographic variable difference on IOTN-AC.

**Table 1:** Main study variables according to gender and monthly household income: Mean (Standard Deviation)

Variables	M (SD)	Gender			Monthly household income				
		Girls	Boys	*F*	< 3000 RMB	3000–5000 RMB	5000–10,000 RMB	> 10,000 RMB	*F*
PIDAQ	1.39 (0.68)	1.51 (0.70)	1.18 (0.57)	24.55^**^	0.94 (0.67)	1.14 (0.54)	1.38 (0.52)	1.81 (0.68)	38.95^**^
IOTN-AC	5.61 (1.86)	5.69 (1.93)	5.46 (1.73)	1.47	5.29 (1.20)	5.84 (1.74)	5.62 (2.04)	5.49 (2.01)	1.37
PSPS-parents	1.99 (1.00)	2.08 (1.05)	1.81 (0.86)	7.07^**^	0.98 (1.27)	5.85 (1.95)	5.20 (1.53)	6.01 (2.02)	14.24^**^
PSPS-peers	1.93 (0.85)	2.00 (0.85)	1.82 (9.83)	4.18^*^	1.73 (0.98)	1.71 (0.74)	1.90 (0.82)	2.41 (1.19)	4.47^**^
PSPS-mass media	1.37 (0.69)	1.39 (0.68)	1.33 (0.72)	0.66	1.25 (0.56)	1.31 (0.60)	1.28 (0.62)	1.54 (0.83)	4.27^**^
PACS	2.04 (1.01)	2.15 (1.01)	1.83 (1.00)	10.25^**^	1.91 (1.02)	1.96 (1.01)	2.07 (0.94)	2.13 (1.05)	1.00

^*^p < 0.05, ^**^p < 0.01

### Testing mediating pathways

Descriptive statistics for the main variables and Spearman correlation coefficients are presented in Table 2. PIDAQ had a moderate to strong association with IOTN-AC, *r* = 0.41, *p* < .01, indicating that poorer dental aesthetics is associated with worse psychological impact. PIDAQ also had significant association with all the three sources of social reinforcement, with moderate association with parents (*r* = 0.39) and peer (*r* = 0.32) sources and week correlation with media (*r* = 0.16) source (all *p*s < .01). Dental appearance social comparison had moderate to small association with three sources of social reinforcement (all *r*s ≥ 0.17, *p*s < 0.01).

**Table 2:** Spearman correlation coefficients between main variables

	1	2	3	4	5
1. PIDAQ	1				
2. IOTN-AC	0.41^**^	1			
3. PSPS-parents	0.39^**^	0.13^**^	1		
4. PSPS-peers	0.32^**^	0.08	0.26^**^	1	
5. PSPS-mass media	0.16^**^	0.07	0.14^**^	0.02	1
6. PACS	0.27^**^	0.06	0.29^**^	0.23^**^	0.17^**^

^**^p < 0.01

According to the results of correlation analyses, all of the three mediating pathways were tested (Table 3). Three sets of analysis showed significant indirect effects of social comparison as a mediator between all three sources of social reinforcement and PIDAQ (Parents-PACS-PIDAQ pathway, *z* = 3.04, *p* = .002, *R*^2^ = .13; Peers-PACS-PIDAQ pathway, *z* = 3.35, *p* < .001, *R*^2^ = .10; Media-PACS-PIDAQ pathway *z* = 2.34, *p* = .019, *R*^2^ = .04). Specifically, after adding teeth social comparison into the pathway, the standard beta weight of social reinforcement on PIDAQ decreased from 0.36 to 0.32 for parent reinforcement (Δ*R*^2^ = .03), 0.31 to 0.27 for peer reinforcement (Δ*R*^2^ = .03), and from 0.21 to 0.17 for media reinforcement (Δ*R*^2^ = .01), respectively (all *p*s < .001).

**Table 3:** Mediational path analyses and Sobel test

	DV	IV	*β*	*R*^2^	Δ*R*^2^
PSPS-parents — PACS — PIDAQ				*R*^2^ (PSPS-parents)	
Step 1: Linear regression	PACS	PSPS-parents	.28^***^		
Step 2: Linear regression	PIDAQ	PSPS-parents	.36^***^	.13	
Step 3: Linear regression	PIDAQ	PSPS-parents + PACS	.32^***^ + .16^***^	.10	0.03
Step 4: Sobel test			*z* = 3.04, *p* = .002		
PSPS-peers — PACS — PIDAQ				*R*^2^ (PSPS-peers)	
Step 1: Linear regression	PACS	PSPS-peers	.21^***^		
Step 2: Linear regression	PIDAQ	PSPS-peers	.31^***^	.10	
Step 3: Linear regression	PIDAQ	PSPS-peers + PACS	.27^***^ + .19^***^	.07	0.03
Step 4: Sobel test			*z* = 3.35, *p* < .001		
PSPS-media — PACS — PIDAQ				*R*^2^ (PSPS-media)	
Step 1: Linear regression	PACS	PSPS-media	.19^***^		
Step 2: Linear regression	PIDAQ	PSPS-media	.21^***^	.04	
Step 3: Linear regression	PIDAQ	PSPS-media + PACS	.17^***^ + .21^***^	.03	0.01
Step 4: Sobel test			*z* = 2.34, *p* = .019		

*DV* Dependent variable, *IV* Independent variable

^***^p < 0.001

### Structural equation modeling

**Fig. 1:**
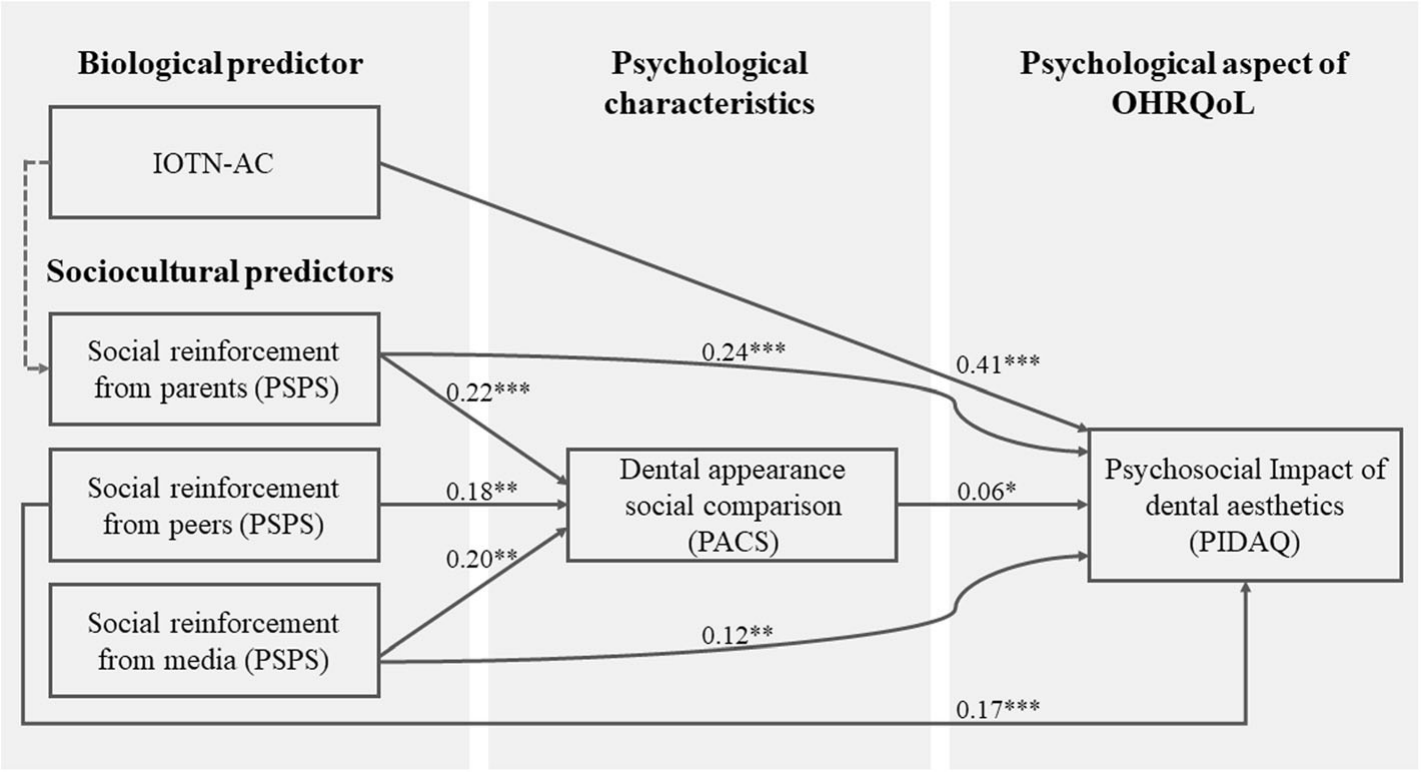
Hypothesized and final model with standardized path coefficients. The pathway from IOTN-AC to social reinforcement of parents in the hypothesized model was not significant, so it was removed in the final model. χ2 = 3.17, df = 4, *p* = 0.529, χ2 /df = 0.79, CFI = 1.000, RMSEA (90% CI) < 0.001 (0.000, 0.066), AIC = 49.17. AIC, Akaike information criterion; CFI, comparative fit index; IOTN-AC, aesthetic component of the index of orthodontic treatment need; RMSEA: root mean square error of approximation. ^*^p < .05; ^**^p < .01; ^***^p < .001

Based on the above observation, one hypothesized model was proposed to investigate the impact of both biological condition (dental aesthetics) and sociocultural factors (social reinforcement from parents, peers and mass media) on psychosocial aspect of OHRQoL (psychosocial impact of dental aesthetics). SEM evaluation showed good fit indices of this model. However, Wald tests and *t*-values indicated that the path from IOTN-AC to parents’ reinforcement failed to attain significance (standard estimate = 0.05, *p* = .253). After deleting this path, SEM evaluating was carried out on the modified model. Results showed that every path was significant and it achieved better fit indices than the hypothesized model (Fig. 1). In this model, children’s dental aesthetics showed direct and strong influence on PIDA. Three sources of social reinforcement had both direct and indirect impact on the criteria variable, and teeth social comparison mediated all of the three indirect influential pathways.

## Discussion

Extending previous multidimensional OHRQoL models, to our knowledge, the current study is the first to evaluate the impact of sociocultural, psychological and biological factors as well as their interaction on orthodontic specific OHRQoL among adolescent patients. Wilson et al., firstly introduced and emphasized sociocultural factors for understanding OHRQoL [3], then different theoretical models for OHRQoL were established [7, 8], which incorporated both clinical and psychosocial factors. Recently, a detailed framework involving environmental factors, sociocultural factors, and psychological factor for understanding OHRQoL was established among adults by Gupta et al., which assessed and emphasized the impact of sociocultural and psychological factors as well as their interaction on OHRQoL [9]. Based on the above works, we further extended these models to orthodontic specific OHRQoL among adolescent patients. A structural equation model was established. As hypothesized, two direct pathways were observed that patients’ dental aesthetics and all three sources of social reinforcement directly predicted PIDA. Meanwhile, we observed indirect pathways, that three sources of social reinforcement predicted PIDA, in part, through dental appearance social comparison.

Consistent with previous findings [36, 37], patients’ dental aesthetics measured by IOTN-AC had robust and unique association with PIDA (*r* = 0.41, *R*^2^ = 16.81%) among adolescents, which meant that poor dental aesthetics predicted poor orthodontic specific OHRQoL. This direct impact was independent of other salient factors. We indeed observed a moderate to weak correlation between IOTN-AC and social reinforcement from parents. However, the structural equation model showed that this hypothesized indirect pathway of IOTN-AC **—**social reinforcement from parents**—** PIDA was not significant. These results, along with previous findings, again provide support for the direct association between dental aesthetics and OHRQoL.

The current study refined the sociocultural factors into three sources, namely the influence from close interpersonal network (parents and peers) and mass media. Most interesting, the three above sources of social reinforcement had both direct and indirect impact on psychological aspects of OHRQoL. Those sociocultural sources, in part, exerted their impact via dental appearance social comparison. Higher perceived pressure from parents, peers and mass media to improve dental appearance predicted poor OHRQoL. These findings are consistent with observations regarding body image concerns from both Western and Asian samples, which linked those social reinforcement as well as appearance comparisons with body dissatisfaction, indicating that the above sociocultural factors had reliable and robust effects on body image concerns [18, 22, 38]. Nevertheless, a particularly novel finding of the current study was the implication of social reinforcement on dental aesthetics and dental appearance social comparison in the prediction of psychological aspects of OHRQoL among adolescent orthodontic patients. Adolescent patients, as early as in the middle school, have already confronted messages about how their teeth should look like partly via social comparison, during which they compared themselves with their friends or models in the mass media to assess whether they meet the attractiveness standards. The current results provide preliminary evidence to support for the hypothesis that social reinforcement of attractive dental appearance in the proximal environment, along with dental appearance comparison, may foster the development of poor OHRQoL in the psychosocial dimension.

Social reinforcement from parents, peers and mass media, in the current study, accounted for 13, 10 and 4% variance of PIDA. After adding dental appearance-related social comparison, these sources of reinforcement accounted for 10, 7 and 3% variance respectively. These coefficients of determination were not high, especially for mass media reinforcement. According to previous literature, 10–20% variance is acceptable in terms of impacts from social reinforcement. A similar study found that perceived social pressure (measured using PSPS) accounted for 19% variance of body dissatisfaction in females and accounted for 14% variance after adding social comparison (measured using PACS). Whereas, the mediational effect of social comparison for males was not significant [22]. In the present study, we did not distinguish effects for girls and boys, which might lead to lower coefficients of determination than those in previous studies [22]. In addition, both direct and indirect effects of social reinforcement from media are weak. One possible explanation is that adolescent patients in this study perceived less pressure on dental appearance from mass media (1.37 ± 0.69) relative to parents (1.99 ± 1.00) and peers (1.93 ± 0.85), which led to less influence on PIDA induced by mass media.

Based on the previous literature [10, 39–41] and current observations, sociocultural and psychological factors need further investigation as possible frameworks for oral health promotion. First, among adolescent orthodontic patients, social reinforcement from parents had the most prominent impact on adolescents’ OHRQoL, then was the impact from peers, and the least was the mass media. To be specific, adolescents who perceived more pressure from their parents and peers on their dental appearance were more likely to engage in teeth social comparison, which in turn resulted in poorer OHRQoL. Thus, adolescents’ parents should try to avoid giving negative comments on dental appearance. Future study is needed to test whether positive feedback and social support from their parents and peers would buffer the negative impact of poor dental health, which would in turn reduce adolescents’ dissatisfaction or concern with their dental appearance, showing positive effects on psychosocial impact of dental aesthetics. Second, it would be useful to test whether social reinforcement predicts OHRQoL change at a follow up time point within a sample whose orthodontic treatment need was not met. This prospective design allows us to detect the casual relation between sociocultural predictors and OHRQoL outcome. Also, to test sociocultural influence in an early adolescent sample can investigate whether the magnitude of the effects vary across developmental periods.

The current findings provide some suggestions to improve the OHRQoL of adolescent orthodontic patients. Firstly, this study would have some implications for policy makers. Previous study showed that most adolescent patients in China seeking orthodontic treatment was to improve their facial appearance [42]. However, orthodontic treatment is paid out of pocket in mainland China, and the delay of orthodontic treatment was mainly due to the family economic conditions [42]. Thus, making corresponding public policies, such as specific insurance products, would provide more opportunities for adolescents with malocclusion to receive orthodontic treatment and in turn would be beneficial for their OHRQoL. Secondly, the current model emphasized the role of social reinforcement on psychosocial dimension of OHRQoL. Although this study only measured reinforcement from parents, peers and mass media, the influence of clinicians’ attitudes during orthodontic treatment might be worth noting, too. Clinicians could pay attention to adolescent patients’ psychological aspects and avoid negative social reinforcement on dental appearance, which would contribute to improvement of OHRQoL.

Limitations of the current study should be noted. Psychosocial dimension of OHRQoL was measured using the translate-and-back-translated version of PIDAQ, which was used in previous study in mainland China [11] and has good validity and reliability in the current study. For future research in mainland China, it will be more appropriate to adopt the Chinese version of PIDAQ [43]. Besides, the items measuring dental-related social comparison were adapted from the Physical Appearance Comparison Scale (PACS) [32]. PACS has been used in Chinese sample before, and it had adequate internal consistency, with Cronbach’s *α* for females being 0.85 and for males being 0.80 [22]. In the current study, the modified instrument also has acceptable internal consistency with Cronbach’s *α* = 0.80.

## Conclusion

In sum, this study provides preliminary evidence indicating that dental aesthetics, social reinforcement and dental appearance comparison are reliable predictors of psychosocial dimension of OHRQoL among adolescent orthodontic patients. It enriches the existing OHRQoL models by introducing the interaction between sociocultural and individual psychological factors. Examining the issue in mainland Chinese samples extends research on OHRQoL features to more culturally diverse groups. Meanwhile, with the growth of Chinese economy, the public pay more attention on aesthetics including dental appearance [44]. In such an environment, many sociocultural factors fueled by China’s beauty economy may affect individual behavior and psychology. This study provides initial tests for certain aspects of these factors (social reinforcement). Other macro-level environment factors contributing to psychosocial impact of dental aesthetics among Chinese youth need future investigations.

## Data Availability

The datasets used and analyzed during the current study are available from the corresponding author on reasonable request.

## Abbreviations

AIC: Akaike information criterion
CFI: Comparative fit index
OHRQoL: Oral-health-related quality of life
PACS: Physical appearance comparison scale
PIDAQ: Psychosocial impact of dental aesthetics questionnaire
IOTN-AC: Aesthetic component of the index of orthodontic treatment need
RMSEA: Root mean square error of approximation
SEM: Structural equation modeling
